# Putative mechanism of a multivitamin treatment against insulin resistance

**DOI:** 10.1080/21623945.2024.2369777

**Published:** 2024-06-27

**Authors:** José Antonio Palma-Jacinto, Edgar López-López, José Luis Medina-Franco, Oreth Montero-Ruíz, Isela Santiago-Roque

**Affiliations:** a Laboratory of Biochemistry and Neurotoxicology, Faculty of Bioanalysis-Xalapa, Universidad Veracruzana, Médicos y Odontólogos S/N Unidad del Bosque, Xalapa, Mexico; b DIFACQUIM Research Group, Department of Pharmacy, School of Chemistry, Universidad Nacional Autónoma de México, Mexico City, Mexico; c Department of Chemistry and Graduate Program in Pharmacology, Center for Research, Advanced Studies of the National Polytechnic Institute, Mexico City, Mexico

**Keywords:** Inflammation, insulin resistance, multitarget, polypharmacology, vitamins

## Abstract

Insulin resistance is caused by the abnormal secretion of proinflammatory cytokines in adipose tissue, which is induced by an increase in lipid accumulation in adipocytes, hepatocytes, and myocytes. The inflammatory pathway involves multiple targets such as nuclear factor kappa B, inhibitor of nuclear factor κ-B kinase, and mitogen-activated protein kinase. Vitamins are micronutrients with anti-inflammatory activities that have unclear mechanisms. The present study aimed to describe the putative mechanisms of vitamins involved in the inflammatory pathway of insulin resistance. The strategy to achieve this goal was to integrate data mining and analysis, target prediction, and molecular docking simulation calculations to support our hypotheses. Our results suggest that the multitarget activity of vitamins A, B1, B2, B3, B5, B6, B7, B12, C, D3, and E inhibits nuclear factor kappa B and mitogen-activated protein kinase, in addition to vitamins A and B12 against inhibitor of nuclear factor κ-B kinase. The findings of this study highlight the pharmacological potential of using an anti-inflammatory and multitarget treatment based on vitamins and open new perspectives to evaluate the inhibitory activity of vitamins against nuclear factor kappa B, mitogen-activated protein kinase, and inhibitor of nuclear factor κ-B kinase in an insulin-resistant context.

## Introduction

The obesogenic environment is a predisposing factor for the development of obesity, such as sedentary lifestyle, urbanization, rural-to-urban migration, hypercaloric food consumption, and physical inactivity. The final step of the interaction of these factors generates pathophysiological mechanism, such as insulin resistance (IR), which is commonly the starting point for the development of more complex diseases, such as type 2 diabetes mellitus, cardiovascular diseases, metabolic syndrome, and breast or colorectal cancer [1]. Chronic consumption of a hypercaloric diet can lead to inflammation and lipid oxidation with excessive accumulation of lipids in adipocytes, myocytes, and hepatocytes [2].

IR starts in adipose tissue with the phosphorylation of insulin receptors caused by the lipo-inflammation process, which is characterized by an increase in the size of adipocytes (excessive lipid accumulation) and metabolic dysfunction (proinflammatory cytokine secretion). Inflammatory pathways in adipocytes and macrophages begin with mitogen-activated protein kinase (MAPK), Junk kinase (JNK) and inhibitor of nuclear factor κ-B kinase (IKK) phosphorylation, followed by nuclear factor kappa B (NF-κB) activation and translocation into the nucleus [3]. Then, proinflammatory cytokines such as tumour necrosis factor-α (TNF-α), interleukin-6 (IL-6), and interleukin-1β (IL-1β) are produced by adipocytes and stimulate changes in the M2 to M1 phenotype of macrophages. Macrophages M1 are characterized by the secretion of proinflammatory cytokines [4,5].

**Figure 1.**
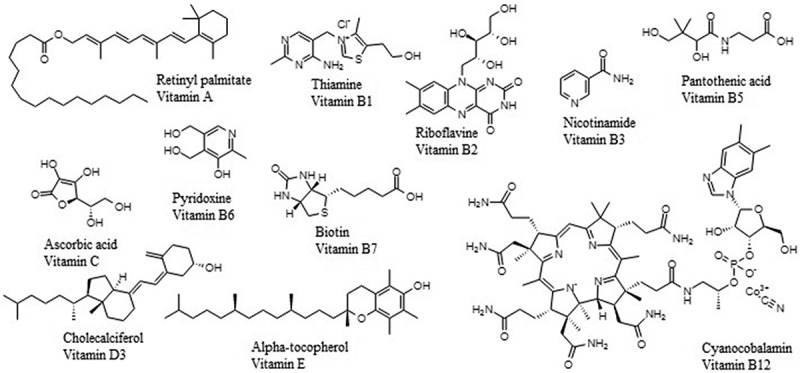
Representative vitamins associated with anti-insulin resistance activity.

Several alternative treatments have been proposed to increase insulin sensitivity and decrease inflammation and oxidative stress levels in obesity models. For example, natural products are promising sources for the development of novel preventive and curative treatments [6–8]. Moreover, vitamins have demonstrated the potential to reduce inflammation and oxidative stress. For example, vitamins A, B1, C, and E have antioxidant activity, and vitamin E can re-establish enzyme glutathione function [9,10]. Vitamins B1 [11], B2 [12], B3 [13], B5 [14], B6, B7, B12 [15], C [16], D3 [17], and E [18] have been shown to have anti-inflammatory activity. Figure 1 shows the structure of exemplary vitamins with anti-insulin resistance activity that have been associated with antioxidant or anti-inflammatory activities, mediated by a decrease in proinflammatory cytokines. However, the precise mechanism of action (MOA) is unclear.

Vitamin B2 shows reduction of TNF-α and other proinflammatory cytokines associated of NF-κB inhibition [12], in the same way, vitamin C administered with vitamin B5 decreased C-reactive protein serum levels [14]. Vitamin C also decreases TNF-α serum levels due to its inhibitory activity on TNF-α mRNA transcription [16], and vitamin E suppresses proinflammatory signalling such as NF-κB and transcription factor STAT3/6 (STAT3/6) [19]. Finally, vitamin D and its receptor (VDR) regulate inflammation by upregulating of MAPK and inhibiting the NF-κB signalling pathway [20]. Therefore, more studies are needed to elucidate the specific MOA of vitamins in the inflammatory pathway. Thus, these molecules represent promising candidates for the treatment or prevention of IR, especially if it is administered a multivitamin (*i.e*., based on a polypharmacy approach or multitarget therapy).

*In silico* studies help to identify promising compounds and molecular targets that represent future solutions for different types of diseases [21–23]. Target prediction using machine learning models, molecular docking calculations, and network pharmacology are examples of the current methodologies used to discover and develop novel drugs [24]. Overall, the rational use of these and other computational techniques saves time, assesses cost-effective drugs, and reduces data gaps [25–27]. Thus, computational methods are powerful for establishing a solid hypothesis for the MOA of the proposed vitamins (A, B1, B2, B3, B5, B6, B7, B12, C, D3, and E) in the inflammatory pathway, which is an important component of IR. Similarly, vitamin D3 was evaluated for its inhibitory activity on mitogen-activated protein kinase kinase 1 (MAPK1) and MAP2K1 by GO and KEGG enrichment analyses. Suggesting that vitamin D3 plays a key role in the prevention of colorectal cancer (CRC) through core targets, the phosphatidylinositol 3-kinase- protein kinase B (PI3K-AKT), hypoxia-inducible factor 1 (HIF-1), and forkhead box protein (FoxO) pathways [28]. In addition, vitamin D3 plays an important role in the inhibition of the NF-κB pathway; therefore, it has been proposed as a treatment for rheumatoid arthritis [29].

The main goal of this work was to describe the putative mechanisms of 11 vitamins with anti-inflammatory activity, inhibiting pathways such as nuclear factor kappa B (NF-κB), inhibitor of nuclear factor κ-B kinase (IKK), and mitogen-activated protein kinase (MAPK), involved in the prevention or treatment of IR. Using bibliographic data analysis and *in silico* methods such as target prediction and molecular docking calculations. *In silico* methods used in this study were validated using both positive and negative controls.

## Results

**Figure 2.**
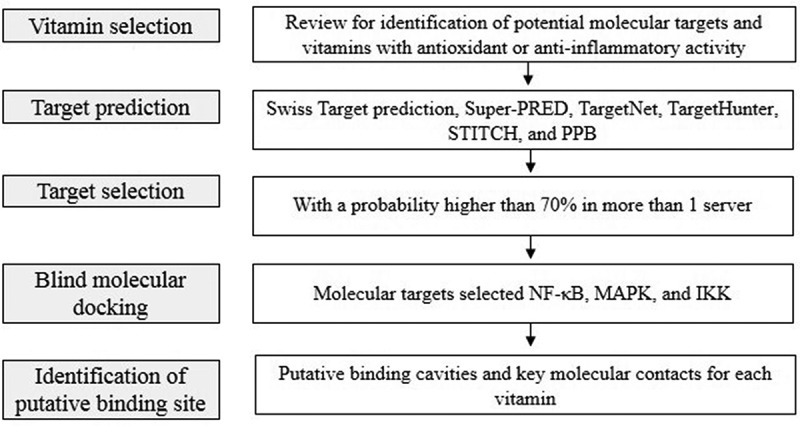
General workflow used in this work.

Using a knowledge-based drug design approach, we explored *in silico* the putative affinity of vitamins A, B1, B2, B3, B5, B6, B7, B12, C, D3, and E with targets involved in the anti-inflammatory potential of the nuclear factor kappa B (NF-κB) pathway. Figure 2 illustrates the general protocol used. *In vitro* and *in vivo* data supported the computational predictions.

### Target prediction

Target prediction is a commonly used technique in drug discovery [30]. The most common target prediction servers allow the identification of potential targets of small molecules based on their structural and/or interactome similarity with each target inhibitor contained in the target prediction servers databases. In this study, we used target prediction servers based on similarities and interactome similarities to identify potential targets for each studied vitamin.

Table 1 and Table S1 in the Supporting Information summarize the interactions of vitamins A, B1, B2, B3, B5, B6, B7, B12, C, D3, and E with NF-κB, MAPK, and IKK using seven target predictor servers (Swiss Target prediction [31], Super-PRED [32], TargetNet, TargetHunter [33], STITCH [34] and PPB [35]). We selected the prediction with a higher than 70% confidentiality and selected for a deep study of the targets predicted in almost two different servers. The benchmark was selected according to the report by Gallo [36], which remarks on the structural diversity of inhibitors of different kinds of targets. Interestingly, approximately 10% of the consensus predictions have been documented in previous studies (Table 1), which indicates the utility of these servers.

**Table 1.** Vitamins target prediction using different servers.

Vitamin	Consensus target prediction	Reference that supports their putative inhibitory activity against each predicted target
A	Tyrosyl-DNA phosphodiesterase 1^2, 6^	-
	MAP Kinase^2,6^	-
	Retinoid acid receptor (alpha, beta, and gamma)^2, 3, 4, 5, 6^	[37]
B1	Transketolase^1, 4, 5, 6^	[38]
	Nuclear factor NF-kappa-B p105 subunit^2^	[39]
B2	Nuclear factor NF-kappa-B p105 subunit^2^	[39]
	MAP kinase^2^	-
	Histone-lysine-N-methyltransferase, H3-lysine-9 specific 3^4, 6^	-
B3	NAD-dependent deacetylase sirtuin 1, 2 and 3^1, 3, 4, 5, 6^	[40]
	Poly [ADP-ribose] polymerase 1^4, 6^	[41]
	Nicotinate phosphoribosyltransferase^5, 6^	-
	NAD-dependent deacetylase sirtuin 1, 2 and 3^1, 3, 4, 5, 6^	[42]
B5	Nuclear factor NF-kappa-B p105 subunit^2^	-
B6	-	-
B7	Insulin-degrading enzyme^1, 6^	-
	MAP kinase^6^	-
	Prelamin-A/C ^4, 6^	-
	Histone-lysine-N-methyltransferase, H3-lysine-9 specific 3^4, 6^	[43]
	Lysine-specific demethylase 4A^4, 6^	-
B12	Nuclear factor NF-kappa-B p105 subunit^2^	[44]
C	Glycogen synthase kinase-3 beta^1, 6^	-
	Arachidonate 15-lipoxygenase^3, 6^	-
	Alpha-amylase^4, 6^	[45]
D3	Dual specificity phosphatase^1, 6^	[46]
	Vitamin D receptor^1, 2, 4, 6^	[47]
	Glycine receptor subunit alpha-1 ^1, 2, 6^	-
	Androgen receptor ^1, 4,^	[48]
	Tyrosyl-DNA phosphodiesterase 1 ^2, 6^	-
	Histone-lysine-N-methyltransferase, H3-lysine-9 specific 3 ^4, 6^	-
E	Glutathione S-transferase Pi^2, 5^	[49]

**Notes**^1^Swiss Target prediction: Probability higher than 70%.

^2^Super-PRED: Probability higher than 90%.

^3^TargetNet: Prediction generate with MACCS, ECFP4, and ECFP6 fingerprints/Probability higher than 90%.

^4^TargetHunter: Probability higher than 0.7.

^5^STICH: Predicted functional score higher than 0.90.

^6^PPB: Calculated with APFp algorithm.

### Molecular docking

**Figure 3.**
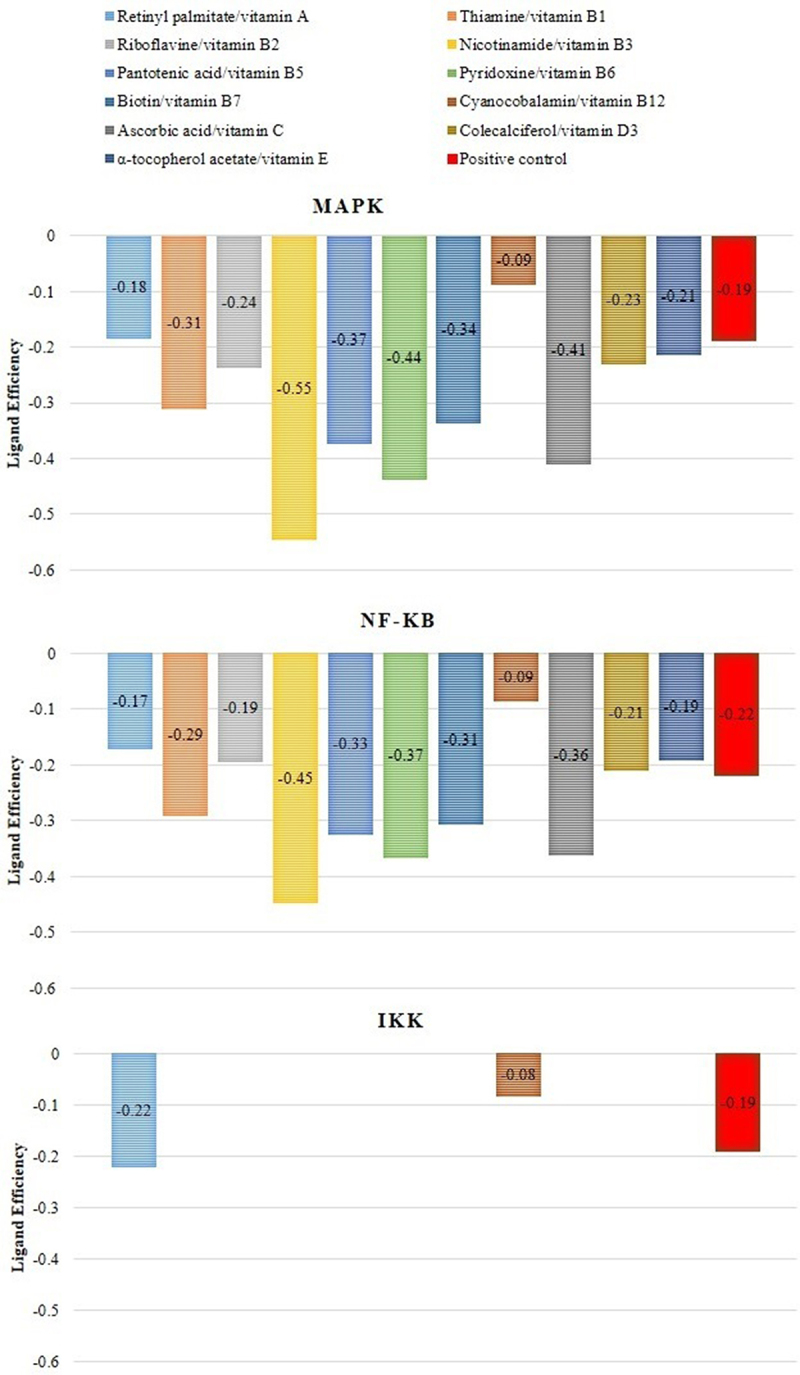
Putative ligand efficiency of vitamins against selected targets related to IR.

**Figure 4.**
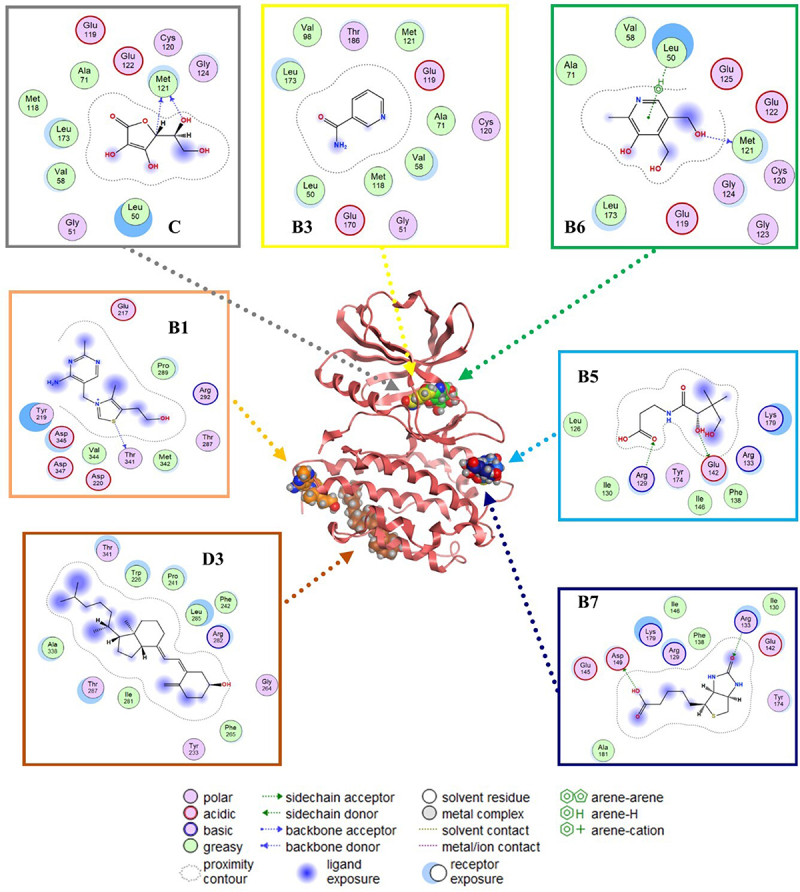
Putative binding sites and molecular interaction predicted by blind molecular docking of vitamins against MAPK.

**Figure 5.**
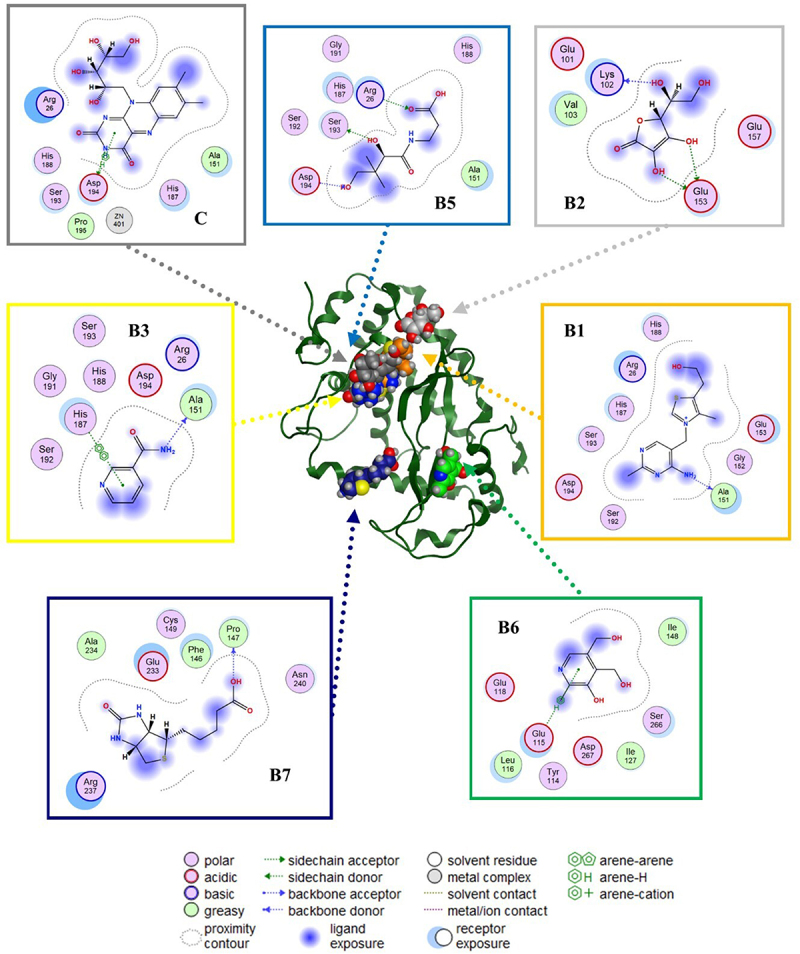
Prediction of binding sites and molecular interactions between vitamins and NF-kB using blind molecular docking.

Molecular docking has been used for drug discovery and optimization [50]. Docking helps to identify putative binding sites, potential inhibitors, and/or identifying new molecular targets, including targets related to IR like inflammatory pathway (NF-κB, MAPK, and IKK) that lead to the proinflammatory cytokine’s translation, and oxidative stress pathway, involved in the phosphorylation of ISR [51,52]. In this case, we used a blind docking approximation to identify the putative binding site (and hypothetical affinity) of each vitamin against targets related to the NF-KB pathway (Figures 3, 4 , and 5). We used positive controls for NF-κB (*i.e*., oxidized hTRX), MAPK (*i.e*., SRC-SM1-71-R), and IKK (*i.e*., Cmpd1/2), and established the molecular docking conditions to reproduce their crystallographic reports with an RMSD value lower than 2 Angtroms (that is, the traditional protocol to validate blind docking protocols). The results of that study established a robust pharmacological hypothesis of protein-vitamin interactions associated with their polypharmacological activity or selectivity against targets related to IR, as illustrated in Figure 3. The polypharmacological activity and selectivity against targets related to IR are illustrated in Figure 3.

In this study, we employed molecular docking calculations to provide insights into the putative affinity of vitamins (A, B1, B2, B3, B5, B6, B7, B12, C, D3, and E for), MAPK and IKK at the structural level.

The most representative binding positions of vitamins B1, B3, B5, B6, B7, C, and D3 against MAPK are illustrated in Figure 4, which shows the putative binding cavities and key molecular contacts. For example, as mentioned previously, vitamins C and D3 can inhibit MAPK [53,54]. Interestingly, the molecular docking results suggest that vitamins B3 and B6 could interact in a similar binding site to vitamin C with similar affinity (Figure 4), highlighting the importance of key interactions with LEU 50 and MET 121, which form part of the protein kinase domain. Vitamin B1 shows a binding site similar to that of vitamin D3, highlighting the importance of THR 341, which forms part of the p38 mitogen-activated protein kinase (p38MAPK)-binding site [55]. However, most of the vitamin B1 and D3 structures do not directly interact with MAPK (solvent ligand exposure), suggesting that their MOA could be generated by promoting allosteric changes in MAPK. In the case of vitamins B5 and B7, their putative binding mechanism could be completely different from that of vitamins C or D3, but in both cases, the positively charged amino acids (*e.g*., ARG, GLU, or ASP) could explain their affinities.

Figure 5 illustrates the predicted binding of the vitamins to NF-κB. As previously mentioned, vitamins B2, B5, and B6 can inhibit NF-κB [15]. The key interactions observed between B2 and B5 against NF-κB highlight the importance of positively charged amino acids (*e.g*., ARG 26, LYS 102, GLU 153, and ARG 194) in their putative binding site, which forms part of the RHD domain involved in the molecular recognition of IKK [56,57]. Similar key interactions with B2 and B5 were predicted for vitamins B1, B3, and C. In contrast, blind molecular docking results suggested that vitamins B7 and B6 interact with different binding sites of the RHD domain in NF-κB.

## Discussion

IR is an uncontrolled pandemic, principally associated with lifestyle. For example, unhealthy alimentary habits promote IR, which are associated with the development of type 2 diabetes mellitus, metabolic syndrome, and cardiovascular diseases. IR is generated initially by inflammation caused by lipid accumulation in the adipose tissue, liver, and muscle, inducing phosphorylation of insulin receptors, followed by insulin resistance. Previous studies have shown that vitamins can prevent IR due to their anti-inflammatory activity and decreased levels of TNF-α, IL-6, IL-1, and IL-1β in the serum [10,12,14,16,19,20]. In the present work, we used an *in silico* workflow (Figure 2) to formulate mechanistic hypotheses at the molecular level regarding the putative anti-inflammatory activity of vitamins against targets related to IR (*e.g*., NF-κB, MAPK, and IKK).

MAPK is the first step in the inflammatory pathway ending in NF-κB activation; this process has been implicated in chronic diseases, such as metabolic diseases [58–60]. Therefore, we considered three important points: NF-κB, MAPK, and IKK. A previous study *in vitro* of vitamin C in HeLa cells showed its inhibitory effects on IKK [61]. A similar effect has been reported for vitamin D [62]. IKK is also inhibited by vitamin E [19].

Interestingly, previous studies have reported the inhibitory activity of vitamins C and D against MAPK, and their indirect inhibition of NF-κB consequently stops the transcription of proinflammatory cytokines [16,20]. In addition, vitamins B2, B5, and B6 inhibit the p105 subunit (of NF-κB), thereby decreasing the levels of proinflammatory cytokines [12,14,63]. These findings were consistent with our blind molecular docking results (Figures 3, 4 , and 5). However, our results highlight the potential inhibitory activity of additional vitamins (*e.g*., B1, B3, B5, B6, and B7) against MAPK or (*e.g*., B1, B3, B7, and C) NF-κB.

IKK, another target of insulin resistance, is associated with vitamin inhibition. For example, vitamins E [19], C [61], and D3 [62] inhibit inflammation and decrease the intracellular proinflammatory state. However, our results did not reproduce these reported interactions. In contrast, our findings highlight the putative interaction between vitamin A and B12 and IKK, and the molecular models that represent these molecular interactions are available in Table S1 in the supplementary material section.

In summary, several studies show that vitamins administered individually (*e.g*. B2 [12], B3 [13], B5 [14], B6, B7, B12 [15], C [64], D [65], E [66]) or co-administrated using multivitamins formulations [67–69] can decrease IR, guided by is the regulation of oxidative stress pathways [70].

Additionally, vitamins show have demonstrated an anti-inflammatory effect by decreasing serum levels of TNF-α, IL-6, IL-1, and IL-1β [10,12,14,16,19,20]. Nevertheless, vitamins C [15], D [65] have been associated with the non-genomic pathway MAPK, vitamins C [61], D3 [62] and E [36] can inhibit IKK and B2, B5, and B6 inhibit the p105 subunit of NF-κB [12,14,63]. In line manner, our main results suggest the potential inhibitory activity of vitamins B1, B3, B5, B6, and B7 against MAPK or B1, B3, B7, and C the potential inhibitory of NF-κB. In contrast, our results did not reproduce inhibitory activity of IKK with previous reports.

In addition, the authors remarked on the limitations of this study. For example, targets with a probability of 70% (in target predictor servers) could be inhibited by vitamins, but this was not considered in this study. In addition, we performed molecular docking with the structures of the vitamins in their pure state and not with their respective metabolites, as we expected to find them in *in vivo* models. Additionally, there is insufficient experimental data to confirm the putative binding site predicted in this study. For this reason, the authors remark on the importance of generating new crystallography, i*n vitro*, and *in vivo* studies to complement this gap in information, allowing the generation of new perspectives and hypotheses. For example, the following section provides an integrative overview of *in silico*, *in vitro*, and *in vivo* achievements that explain the complex context of IR in more detail, which could lead to the rationalization of a novel polypharmacology therapy to combat it.

### Overview and perspectives

**Figure 6.**
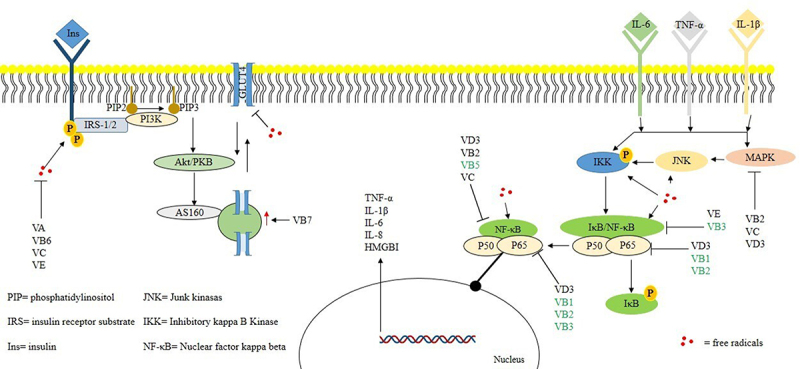
Summary of vitamins and targets validated on in vivo or in vitro models and putative inhibitory activities (predicted with in silico tools in this work).

Inflammation is a key endpoint of IR, and vitamins playing a significant role in IR protection [8]. For example, several vitamins (*e.g*., B1, B2, B3, B5, C, D3, and E) have the potential to restore the inflammatory environment in metabolic diseases [64–66] and mediate the regulation of targets related to inflammatory pathways (*e.g*., MAPK, IKK, JNK, and NF-κB; Figure 6).

Several researchers have reported that the administration of a multivitamin complex can prevent IR [67,68,71–73]. Multivitamin treatment significantly decreased the serum levels of TNF-α, IL-6, IL-1, and IL-1β in murine models. However, anti-inflammatory related targets (such as NF-κB, MAPK, and IKK) have not been quantified, generating an information gap in the complete understanding of the anti-inflammatory mechanisms [20]. This information limitation justifies our work, which improves our knowledge about the anti-inflammatory effects of vitamins.

Polypharmacology offers new perspectives that can resolve different issues related to single-target drug design [26,74]. Simultaneously, polypharmacology presents the possibility of generating novel and refined approaches to address complex diseases such as metabolic diseases (*e.g*., IR) [75,76]. In this context, the generation and optimization of polypharmacological formulations represent a new challenge in drug design. Which is imperative for the study of their interaction with secondary targets. Furthermore, the experimental validation (using *in vitro* models) of multiple target interactions does not guarantee their clinical relevance; in these cases, the clinical impact must be confirmed using *in vivo* models.

This study provides an overview of vitamins with potential applications in IR treatment and prevention. Vitamins were selected based on previously reported knowledge. Furthermore, this study was limited by the selected targets (Figures 2, 3 , and 4). However, this is a key point in the design of new chemical entities against IR and a discussion point regarding its nutraceutical role.

Moreover, this bibliographic and *in silico* study (using validated methods) uncovers the possibility of identifying vitamins that act at different molecular levels (*i.e*., changing the signalling, gene expression, epigenetic modifications or physiological effects) on IR, and opens new hypotheses that must be corroborated by *in vitro* or *in vivo* models. In fact, the parallel modulation of different endpoints could contribute to reducing the necessary doses to generate therapeutic effects that simultaneously contribute to reducing the associated side effects in *in vivo* models and the excessive economic costs derived from it. In addition, it can be used as an alternative to animal models. Finally, understanding the polyactivities of vitamins could improve preventive medicine [77–79].

## Conclusions

The role of vitamins in the regulation of the NF-κB signalling pathway during IR, and this regulation was associated with MAPK, IKK, and NF-κB, without neglecting free radicals, such as important mechanisms involved in insulin receptor oxidation and indirect activation of the NF-κB pathway.

Our main results indicated that vitamins C and D3 interact with the protein kinase domain and p38 MAPK-binding site of MAPK. Additionally, the results suggest that vitamins B2, B5, and B6 interact with the rel homology domain (RHD) domain of NF-κB, which explains their inhibitory activity against MAPK and NF-κB. Finally, the molecular docking results suggested that vitamins A and B12 protect against IKK. The present work clearly opens new perspectives to study targets such as NF-κB, MAPK, and IKK involved in the inflammatory context of IR and in the development of multivitamin treatments. A follow-up study could discuss the regulation of the NF-κB signalling pathway, MAPK, and IKK in the prevention or treatment of IR, which must be investigated *in vitro* and *in vivo* based on drug discovery.

## Methodology

The methodology includes diverse *in silico* tools to improve our understanding of the putative polypharmacological activity of different vitamins (A, B1, B2, B3, B5, B6, B7, B12, C, D3, and E) against possible targets. The first part of the methodology describes the use of predictive servers based on molecular similarity and biological interactome similarity methods to predict the targets of the different vitamins evaluated in this study. The second part of the methodology describes the molecular docking protocol to describe the putative binding interactions between vitamins and the main targets (NF-κB, MAPK, and IKK) predicted in the first methodological section.

### Target prediction

Each vitamin was represented using the canonical SMILES obtained from the ChEMBL database, version 33 [80]. Each SMILE code was used to predict putative interactions of each vitamin with different targets. We used seven target-predictor servers (Swiss Target prediction [31], Super-PRED [32], TargetNet, TargetHunter [33], STITCH [34] and PPB [35]) based on structure similarity and biological interactome similarity respect to reporter inhibitors. Targets related to IR with *in vitro* and *in vivo* validation, which additionally have been predicted by (at least) two different servers, were studied using molecular docking (*vide infra*).

### Molecular docking

#### Protein and ligand preparation

The crystallographic structures of MAPK, NF-κB, and IKK (PDB IDs: 3FHR, 4Q3J, and 4E3C, respectively) were retrieved from the Protein Data Bank (available online: https://www.rcsb.org/ (accessed on 12 March 2023) [81]. The ligands were built and energy-minimized in the Molecular Operating Environment (MOE) software v. 2023 using the MMFF94× force field [82]. Protomers that were more stable at physiological pH were identified.

#### Molecular Operating Environment

The MOE software was used to generate the docking conformation of the protein-ligand complexes. For MAPK, the grid was centred in the inhibitor cavity, with a size of 27 Å^3^. For NF-κB and IKK, a grid was constructed around the complete proteins, that is blind docking [27]. Using the ‘Triangle Matcher’ method, the binding compounds were subjected to 50 search steps (poses) and the default values for the other parameters. Clusters with RMSD < 2 Å were visually explored and considered representative and potential interactions against the studied targets [83]. During docking simulations, the receptor was considered rigid, and the ligands were flexible. Conformations with the lowest binding energy were selected for additional and exhaustive visualization. For each target, the molecular docking protocol was validated, and the binding pose of crystallographically reported inhibitors was reproduced using this protocol. For example, for NF-κB, MAPK, and IKK, we used oxidized hTRX, SRC-SM1-71-R, and Cmpd1/2 as positive controls. Crystallographic poses were reproduced with an RMSD value lower than 2 angstroms [84–86].

### Ligand efficiency calculation

Ligand efficiency (LE) is used to improve the interpretation of docking scores because it affects the size of the ligand, which can have a marked influence on the binding sites [87]. The LE was calculated from the docking score of each control and ligand (*i.e*., vitamins) using Equation 1:

(**1**) Ligand Efficiency = Docking Score/Heavy Atom Count

The Heavy Atom Count for each ligand was calculated using DataWarrior software V. 5.5.0 [88].

## Abbreviation list

**Table:** 

CRC	colorectal cancer
FoxO	forkhead box protein
HIF-1	hypoxia-inducible factor 1
IKK	inhibitor of nuclear factor κ-B kinase
IL-1β	interleukin-1β
IL-6	interleukin-6
IR	insulin resistance
JNK	Junk kinase
LE	ligand efficiency
MAPK	mitogen-activated protein kinase
MAPK1	mitogen-activated protein kinase kinase 1
MOA	mechanism of action
MOE	molecular operating environment
NF-κB	nuclear factor kappa B
p38MAPK	p38 mitogen-activated protein kinase
PI3K-AKT	phosphatidylinositol 3-kinase-
RHD	rel homology domain
RMSD	root mean square deviation
STAT3	transcription factor STAT3
STAT6	ranscription factor STAT6
TNF-α	tumor necrosis factor-α
VDR	vitamin D receptor

## Data Availability

The data that support the findings of this study are openly available in figshare at http://doi.org/10.6084/m9.figshare.24547936, reference number [89].
